# Benign peripheral nerve sheath tumors: an interdisciplinary diagnostic and therapeutic challenge

**DOI:** 10.1007/s10143-023-02107-z

**Published:** 2023-08-18

**Authors:** Anne-Kathrin Uerschels, Nora F. Dengler, Mehdi Chihi, Annika Lenkeit, Thiemo F. Dinger, Ramazan Jabbarli, Ulrich Sure, Tim Hagenacker, Karsten H. Wrede, Oliver Gembruch

**Affiliations:** 1 Department of Neurosurgery and Spine Surgery, University Hospital Essen, University of Duisburg-Essen, Essen, Germany; 2 Center for Translational Neuro- & Behavioral Sciences (C-TNBS), University Hospital Essen, University of Duisburg-Essen, Essen, Germany; 3 Department of Neurosurgery, Charité - Universitätsmedizin Berlin, Berlin, Germany; 4 Department of Neurology, University Hospital Essen, University of Duisburg-Essen, Essen, Germany

**Keywords:** Benign peripheral nerve sheath tumors, Diagnostics, Mistreatment, Misdiagnosis

## Abstract

A benign peripheral nerve sheath tumor (bPNST) is a rare lesion associated with peripheral nerval structures. Symptoms may be heterogeneous, complicating diagnosis finding. Additionally, management concepts of bPNST may vary. In some cases, initial misdiagnosis leads to mistreatment resulting in severe functional deficits and chronic pain syndromes. Therefore, we analyzed patients treated for bPNST in our specialized institution with a primary focus on prior misdiagnosis and possible mistreatment. Patients with bPNSTs (schwannomas, neurofibromas, hybrid nerve sheath tumors, and perineuriomas) treated at the Neurosurgical Department between January 1, 2015, and July 31, 2021, were included. Assessment of demographics, tumor entity, tumor location, symptoms, the interval between the onset of symptoms and surgery, involved medical specialties, and outpatients’ treatment, with particular focus on initial misdiagnosis and inappropriate medical treatment, was performed. Eighty-five patients were included in the final analysis with schwannoma being the most prevalent histopathological diagnosis (schwannoma (75.3%, *n*=64), neurofibroma (12.9%, *n*=11), hybrid nerve sheath tumor (5.9%, *n*=5), and perineurioma (5.9%, *n*=5)). An incorrect primary diagnosis was detected in 44.7% (*n*=38), leading to suboptimal or insufficient treatment in these cases. Of those, 28.9% (*n*=11/38) were treated suboptimal, while 18.5% (*n*=7/38) underwent unnecessary invasive diagnostics. Inappropriate surgery based on prior misdiagnosis, which led to severe neurological deficits in all these cases, was reported in 26.3% (*n*=10/38). For the first time, our data shows the quantity and impact of incorrect initial diagnosis in bPNST causing a delay in causative treatment or resulting in unnecessary or potentially harmful treatment.

## Introduction

A benign peripheral nerve sheath tumor (bPNST) is a rare lesion of neuroectodermal origin, directly associated with peripheral nerve structures [1]. Clinical presentation of bPNSTs includes asymptomatic palpable lesions, painful palpable lesions, and lesions accompanied by neurological deficits [2–5]. The most common bPNSTs are schwannomas and neurofibromas. Their localization can cause various symptoms such as local swelling, motor deficits, hypoesthesia, and neuropathic pain due to the ongoing pressure on the unaffected nerve fascicles or the loss of function of the affected fascicles themselves. These symptoms can sometimes be misinterpreted as radicular symptoms or other frequently symptomatic conditions, such as joint and muscle pain without a specific cause [2, 3, 6–8].

Diagnostic workup includes neurological examination, magnetic resonance imaging (MRI), ultrasound, and electrophysiological testing. Especially high-resolution nerve sonography is more and more commonly used for peripheral neuropathies [9–12].

However, one major symptom may be a local swelling or a palpable lump that may lead some clinicians to make wrong assumptions regarding tumor origin when careful neurologic assessment is not performed. Differential diagnosis of local swelling includes pathologic lymph nodes, metastasis, soft tissue tumors of non-nervous origin, and others [8, 13].

Patients with the symptom of local swelling may be transferred to different medical subspecialties leading to various diagnostic and therapeutic pathways. For example, neurologists, orthopedics, plastic and reconstructive surgeons, general surgeons, thoracic surgeons, and neurosurgeons may be involved in the treatment of soft tissue masses.

A nervous origin might not be assumed initially, leading to treatment according to the respective guidelines. Consequently, the initial diagnosis and treatment may not be performed by someone experienced in treating benign peripheral nerve lesions, which promotes delayed diagnosis and nerve damage [14].

We aimed to evaluate the rate of primary misdiagnosis and mistreatment in patients suffering from deep-seated bPNSTs. Furthermore, we aimed to identify causative factors for mistreatment.

## Methods

### Study design

A retrospective analysis of our prospective dataset “peripheral nerve lesion” was performed for patients treated at our institution between January 1, 2015, and July 31, 2021.

**Fig. 1:**
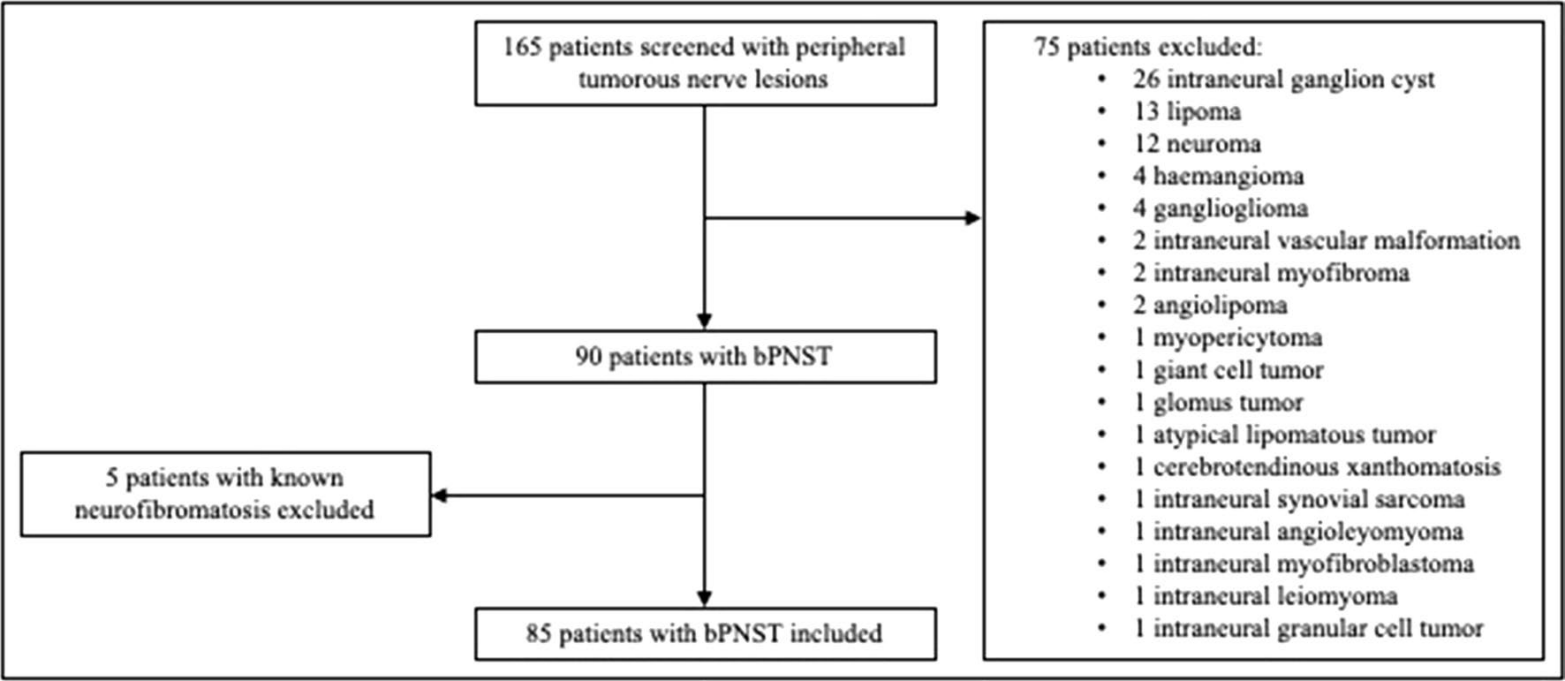
Flow-chart: inclusion and exclusion of patients. bPNST, benign peripheral nerve sheath tumor

Patients with sporadic, deep-seated bPNSTs (schwannomas, neurofibromas, hybrid nerve sheath tumors (HNST), and perineuriomas) involving the extremities and the lumbar or cervical nerve plexus were included. Therefore, patients with cutaneous tumors were excluded from analysis. Furthermore, patients with other benign intraneural tumors without nerve sheet origin and malignant nerve sheath tumors and patients with known neurofibromatosis were also excluded (Fig. 1).

### Ethics

The study was conducted following the STROBE guidelines after approval by the Institutional Review Board (Medical Faculty, University of Duisburg-Essen, Registration number: 18–7955-BO).

### Evaluated parameters

Demographics, tumor entity, tumor location (upper and lower extremity, lumbar or cervical nerve plexus), symptoms, the interval between the onset of symptoms and surgery, involved medical specialties, outpatients’ treatment, diagnostic before surgery (MRI, computed tomography (CT), ultrasound and electrophysiology), possible false diagnosis, and inappropriate medical treatment and mismanagement were evaluated.

### Definition of “misdiagnosis”

The Department of Neuropathology confirmed the final diagnosis of bPNST after surgical treatment at the Department of Neurosurgery and Spine Surgery.

A misdiagnosis occurred, if the final diagnosis was inconsistent with the diagnosis which was set up by the doctor who treated the patient at first. The assessment of misdiagnosis was made based on the external treatment records and each patient’s medical history.

Preoperative assumption of a bPNST different from the final diagnosis was not defined as misdiagnosis.

### Definition of “suboptimal treatment” and “inappropriate surgical treatment”

Suboptimal treatment was assumed in symptomatic patients, if symptoms remained the same or progressed for more than 6 months under conservative treatment and without surgical treatment.

Inappropriate surgical treatment was present, if the patient was surgically treated not due to the bPNST, but because of an accompanying disc prolapse or a nerve entrapment syndrome, for example, without relieve of the symptoms.

Mistreatment was present, if the surgical treatment of the bPNST did not follow the current treatment standard for deep-seated nerval tumors (microsurgical tumor resection with neurophysiological monitoring) [15].

### Statistical analysis

Data were analyzed using SPSS 25.0 (Statistical Package for the Social Sciences, SPSS Inc., Chicago, IL, USA). Metric data were described by mean, standard deviation, and nominal data by frequency and valid percent. *p*-values <0.05 in two-sided testing were considered significant.

Demographic, clinical, and radiographic parameters were analyzed univariately regarding their association or correlation with misdiagnosis or suboptimal treatment and malpractice. Therefore, Pearson’s *χ*^2^ statistics or Fisher’s exact test was used for dichotomous variables.

## Results

### Study population

Over the observed period, 165 patients with peripheral nerve tumors were surgically treated at our department by one neurosurgeon specialized in peripheral nerve surgery. Of those, 80 patients were excluded as they did not meet the inclusion criteria. Our final analysis included 85 patients (44 males, 51.8%) with a mean age of 49.4 ± 16.1 years (range: 13–84 years). Time from the beginning of the symptoms until surgery was 22.9 ± 42.6 months, ranging from 0 months to 348 months (Table 1).

**Table 1:** Patients’ demographics and tumor characteristics

Demographics	
Age (years)	49.4 ± 16.1
Sex	
Females	41 (48.2%)
Males	44 (51.8%)
Tumor entities	
Schwannoma	64/85 (75.3%)
Neurofibroma	11/85 (12.9%)
Perineurioma	5/85 (5.9%)
Hybrid nerve sheet tumor	5/85 (5.9%)
Tumor location	
Upper limb/cervico-brachial plexus	33/85 (38.8%)
Lower limb/lumbo-sacral plexus	46/85 (54.1%)
Other location	6/85 (7.1%)
Presenting symptom	
Pain only	21/85 (24.7%)
Palpable mass and self-induced pain	14/85 (16.5%)
Palpable mass only	15/85 (17.6%)
Neurological deficits	22/85 (25.9%)
By chance	13/85 (15.3%)
Hoffmann Tinel’s sign	50/85 (58%)
Symptom duration until surgery (months)	22.9 ± 42.6

### Tumor entity and tumor location

Histopathological analysis revealed 64 schwannomas (75.3%), 11 neurofibromas (12.9%), five HNSTs (5.9%), and five perineuriomas (5.9%). Most cases affected the lower limb and lumbosacral plexus (54.1%, *n*=46) or the upper limb and cervicobrachial plexus (38.8%, *n*=31). Five bPNTs involved the thoracic nerves and one the supraorbital nerve (Table 1).

### Presenting symptoms

A palpable symptomless mass, discovered by self-examination, was present in 17.6%. Pain as the presenting symptom was described in 24.7%, whereas in 16.5%, a self-induced pain by touching the palpable mass was complained. In 58%, a Hoffmann Tinel’s sign could be triggered by touching or tapping. Neurological deficits such as hypoesthesia, dysesthesia, or motor deficits were detectable in 25.9%. Of those, 86.3% complained about sensory deficits, while 13.6% showed motor deficits. In 15.3%, the tumor was diagnosed by chance during another examination (Table 1).

### Physicians consulted

The patients visited a general practitioner at first in 84.7%, while 5.9% went to a neurologist. Additionally, 5.9% presented to an orthopedic, and 3.5% chose their gynecologist as their primary consultant.

Further referral to a specialist was generally based on the location of the mass (extremities, abdomen, and pelvis) or the leading symptom (neurological deficits, pain). Thus, referral to a general surgeon was made in 16.5%, while 21.2% were referred to an orthopedic based on their symptoms. Furthermore, 11.6% were transferred to a neurologist. An otolaryngologist was visited in 3.6%. However, the majority of 42.4% were transferred to a neurosurgeon (Table 2).

**Table 2:** Overview of the involved medical society and the diagnostics

Physicians consulted at first	
General practitioner	72 (84.7%)
Neurologist	5 (5.9%)
Orthopedics	5 (5.9%)
Gynecologist	3 (3.5%)
Referral to specialist	
Neurosurgeon	36 (42.4%)
Orthopedics	18 (21.2%)
General surgeon	14 (16.5%)
Neurologist	11 (12.9%)
Otolaryngologist	3 (3.6%)
Others	3 (3.6%)
Diagnostic workup	
MRI	82 (96.4%)
CT	1 (1.2%)
Ultrasound	1 (1.2%)
Clinical examination	1 (1.2%)
MRI according to the symptoms	
Palpable mass	14/15 (93.3%)
Palpable mass and self-induced pain	13/14 (92.9%)
Pain	21/21 (100%)
Neurological deficits	21/22 (95.5%)
By chance	13/13 (100%)

*MRI* magnetic resonance imaging, *CT* computed tomography

### Diagnostic imaging

Magnetic resonance imaging as the primary diagnostic tool was performed in 96.4%. However, MRI was often initially focused on the spine, especially in cases of unclear radiating pain as primary symptom (31.7%). There was only one case where a CT scan was used as the initial diagnostic tool and one case where ultrasound was used at first (Table 2).

### Misdiagnosis

An initial incorrect diagnosis was made in 44.7% of patients. In most of the cases, typical spinal disorders were suspected (34.2%), such as spinal canal stenosis or herniated disc of the cervical spine (46.2%) or the lumbar spine (53.8%). A sarcoma was suspected in 23.7%, while a nerve entrapment syndrome was wrongly diagnosed in 13.2% and a malignant lymph node in 10.5%. The other cases (18.4%) consisted of a shoulder-arm syndrome, a malignant peripheral nerve tumor, an idiopathic foot drop paresis, a dental root problem, and a hallux valgus (Table 3).

**Table 3:** Type of misdiagnosis and mistreatment

Misdiagnosis	Suspected diagnosis	Number of Cases
	Cervical spine syndrome	6/38 (15.8%)
	Lumbar spine syndrome	7/38 (18.4%)
	Sarcoma	9/38 (23.7%)
	Nerve entrapment syndrome	5/38 (13.2%)
	Malignant lymph node	4/38 (10.5%)
	Others	7/38 (18.4%)
	Total amount of cases	38/85 (44.7%)
Mistreatment	Prolonged conservative treatment with irreparable damage	11/38 (28.9%)
	Unnecessary invasive diagnostic (CT-guided biopsy)	7/38 (18.5%)
	Wrong surgical treatment based on misdiagnosis	10/38 (26.3%)
	Inadequate surgical technique used	10/38 (26.3%)

### Suboptimal treatment or inappropriate surgical treatment

Prolonged conservative treatment was present in 28.9%, resulting in pronounced neurological handicaps in two cases and neuropathic pain syndrome in five patients.

In 18.5%, an unnecessary invasive diagnostic (CT-guided biopsy) was performed before the definitive therapy due to a suspected diagnosis of a malignant retroperitoneal tumor in a general surgery department. Two patients persistently suffered from neuropathic pain after a CT-guided biopsy, but we did not find any case with permanent neurological deficit related to these biopsies. In all these cases, the biopsy confirmed the correct diagnosis.

Furthermore, an unnecessary surgical treatment unrelated to the bPNST was performed in 26.3%. These were carpal tunnel release, cubital tunnel release, hallux valgus surgery, cervical disc surgery, dental surgery, spinal neurinoma surgery, and shoulder arthroscopy.

Surgical removal or partial removal of the bPNST was performed using an inappropriate surgical technique (non-microsurgical and without intraoperative electrophysiological testing) in 26.3%. This led to severe motor and sensory deficits in seven patients and resulted in revision surgery in every case (Table 3).

### Misdiagnosis and inappropriate surgical treatment and their correlation

Misdiagnosis and mistreatment showed a significant correlation with the treating specialist. Patients referred to a neurosurgeon by the general practitioner under the suspected diagnosis of a bPNST showed significantly lower rates of misdiagnosis and mistreatment than the other specialists (*p*<0.001).

Furthermore, patients with neurological deficits were misdiagnosed and mistreated significantly more often than patients without neurological deficits (*p*=0.003, *p*<0.001, respectively).

Patients receiving MRI of the cervical spine or the lumbar spine as an initial diagnostic tool showed significantly higher rates of misdiagnosis and mistreatment in comparison to patients receiving MRI of another area, which was usually the tumor-bearing region (*p*-value for MRI of the cervical spine: 0.039 for both; *p*-value for MRI of the lumbar spine: 0.010 for both).

Furthermore, MRI of the cervical spine was significantly more often performed in patients with neurological deficits than in patients without neurological deficits (*p*=0.007). This significant difference was not detected for patients receiving MRI of the lumbar spine (*p*=0.275) (Table 4).

**Table 4:** Univariate analyses correlating misdiagnosis and mistreatment with clinical and radiographic parameters

	*p*-value	
	Misdiagnosis	Mistreatment
Referred to (neurosurgeon vs. another specialist)	<0.001	<0.001
First symptom (with vs. without neurological deficit)	0.003	<0.001
MRI cervical spine (present vs. absent)	0.039	0.039
MRI lumbar spine (present vs. absent)	0.010	0.010
	MRI (cervical spine)	MRI (lumbar spine)
First symptom (with vs. without neurological deficit)	0.007	0.275

*vs*. versus

### Illustrative case

**Fig. 2:**
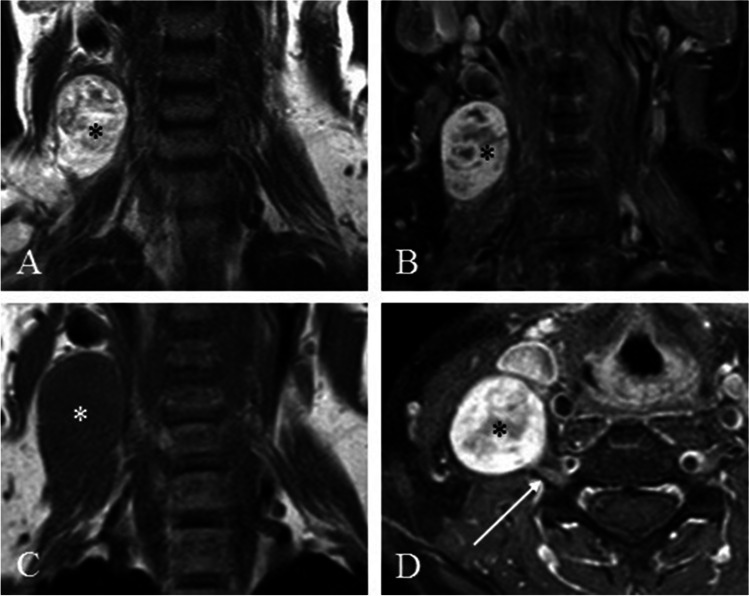
Illustrative case showing the preoperative MRI of the schwannomma (*) misdiagnosed as malignant lymph node resulting in a biopsy. **A** T2-weighted MRI without contrast showing well encapsulated tumor (*), **C** T1-TSE weighted MRI without contrast, **B**, **D** T1-weighted MRI with contrast enhancement of the tumor (*) in direct contact to the CX nerve root (→)

A 52-year-old female patient noticed an elastic swelling on the right side of her neck. After consultation, her general practitioner indicated an MRI of the neck. It showed a smoothly circumscribed paired-spinous mass with apparent contrast enhancement. The diagnosis of “lymph node metastasis” was proposed by the radiologist in charge. Treatment in an Otolaryngologist Department and tumor removal of the mass followed. Intraoperatively, the C6 root was cut for total tumor removal. Histopathological examination revealed the diagnosis of schwannoma. Postoperatively, there was a severe weakness of arm flexion with denervation of the biceps muscle, a disturbance of the fine motor function of the hand, a loss of sensitivity in the area supplied by the C6 root, and a pronounced neuropathic pain in the entire right arm. The patient was referred to our department eight months after surgery. A sural nerve graft was performed, and the patient partially recovered. At last follow-up, seven months after surgery, the patient presented with a weakness of the biceps muscle, the fist, and hypoesthesia of the hand. However, the neuropathic pain symptom entirely disappeared (Fig. 2).

## Discussion

A bPNST is a lesion directly associated with peripheral nervous structures and of neuroectodermal origin [1]. Symptom presentation varies depending on their localization, including local swelling, motor deficits, hypoesthesia, and pain [2, 3, 6–8].

A nervous origin might not be assumed at first due to its rarity, leading to treatment under a suspected different diagnosis. Consequently, misdiagnosis and resulting suboptimal or insufficient treatment with possible severe complications might occur.

For the first time, our study presents data on rates of initial misdiagnosis and mistreatment in patients with bPNST. In our series, 44.7% of patients with sporadic bPNST presenting to our specialized institution had an initial misdiagnosis that led to inappropriate surgical treatment in most of these patients (71.1%).

### Symptom presentation

Thus, in our group of patients, we could find extremely painful, completely painless large, and very small tumors. Hypoesthesia was also commonly reported. The literature only rarely mentions severe or pronounced symptoms such as paralysis or complete loss of sensitivity, and we also found this confirmed by our patients [2, 4]. Some individuals reported about radiating pain to the corresponding extremities, which may explain the relatively high number of spinal MRI. Symptom presentation of perineurioma differs from other bPNSTs, presenting as a mononeuropathy of gradual onset and slow progression, resulting in progressive neurological deficits like hypoesthesia or, much more frequently, a muscular weakness but, at least in our patients, never a positive Hoffmann Tinel’s sign [10, 11]. In 58% of the other patients, a Hoffmann Tinel’s sign was triggered by touching or tapping. This corresponds with the information in the literature that the Hoffmann Tinel’s sign is highly suggestive for a bPNST and should lead to further diagnostics [15–17].

### Diagnosis of bNPST

Diagnostic workup of a bPNST includes MRI, neurosonography, and electrophysiology. MRI is obligatory to confirm or exclude the suspected diagnosis [13, 18]. While MRI of the corresponding region was performed for visible and/or palpable tumors of the extremities, MRI of the spine was performed more frequently for symptomatic but not visible tumors or in cases of radiating symptoms.

In our study, MRI of the spine was performed in all patients with tumors of the sciatic nerve, although they consistently reported triggerable pain. For example, pain was reproducible while sitting on the edge of a hard chair. Consequently, a non-pathological MRI of the spine often led to a delay of definitive diagnosis, especially if conservative therapies were first initiated.

Even though neurosonography is a quick and straightforward method, especially because the nerval tumor origin can be quickly recognized, this method was used only in one case as an initial diagnostic tool. Modern high-resolution nerve sonography makes it possible to visualize the carrier nerve in detail. An experienced examiner can easily make a diagnosis of bPNST. The technique of high-resolution nerve sonography is used in many neurological centers [14, 19].

The situation can be more difficult in cases of retroperitoneal tumors. In those mostly asymptomatic cases, the diagnosis of a retroperitoneal mass as an incidental finding depends on a focused examination of specialized consultants.

Differential diagnosis includes retroperitoneal sarcoma, liposarcoma, or tumors of ovarian origin. Therefore, these patients are often treated appropriately in specialized sarcoma or gynecological centers. A CT-guided biopsy is performed before therapy planning as an additional diagnostical tool according to their guidelines.

### Causes of misdiagnosis

We were able to identify four main causes resulting in misdiagnosis of bPNST. Firstly, variation of different symptoms and especially failure to recognize and interpret specific symptoms (for example, a positive Hoffmann Tinel’s sign) may lead to misdiagnosis concerning a non-pathological MRI of the spine. Practitioners might then stop further diagnostic workup.

Secondly, symptom presentation of bPNST might be similar or mimic symptoms of more common diseases. In particular, symptoms similar to radicular symptoms (pain radiating to an extremity) caused by degenerative spinal disorders and symptoms similar to those of nerve entrapment syndromes need to be mentioned. In our study, spinal MRI was often performed in cases of radicular pain. Misdiagnosis was significantly higher in those patients. Therefore, practitioners might be misguided by the presenting symptoms.

Thirdly, bPNST may occur in every region of the body. Radiologists might interpret findings according to their professional experience and knowledge and the frequency and probability of the presented findings. Therefore, one might diagnose a cervical disc prolapse in cases with radiating pain, a malignant lymph node in the axilla or the neck, or a retroperitoneal sarcoma instead of a rare bPNST. In our study, bPNST of the brachial or lumbar plexus were misdiagnosed as malignant lymph nodes or sarcoma. In those cases, anatomical relation to the plexus was not detected or misinterpreted; therefore, bPNST as a differential diagnosis was neglected. While the incidence of bPNSTs in patients with neurofibromatosis type I is well studied, usable results on the frequency of sporadic bPNST do not exist in detail. In literature, the incidence of 10–15% of treated soft tissue tumors is often quoted [2, 3, 6–8].

Lastly, in the German medical system, general practitioners are the first consultants of the patients and are essential for organizing further therapy at a specialized center (84.7% in our study). Therefore, they significantly impact treatment with their “gatekeeper function.” Furthermore, diagnosis and therapy depend on a specialized consultant. We could show that patients referred to a neurosurgeon had significantly lower rates of misdiagnosis and mistreatment. However, it is understandable that medical specialization leads to an influenced interpretation of findings due to the low frequency of diagnoses made in the corresponding field [20–22].

### Causes of mistreatment

Our data show the prolonged time for a symptomatic patient to receive a definite diagnosis and adequate therapy in many cases. Failure to recognize a nerve tumor as the cause of pain or sensation deficits leads, at best, only to delayed therapy or, in more serious cases, to the development of a chronic pain syndrome or severe neurological deficits [14, 23]. However, chronic pain and the missing explanation of individual symptoms can lead to a significant psychological burden [24]. Additionally, patients with neurologic deficits were at particular risk for misdiagnosis and mistreatment. Moreover, MRI of the spine did not prevent misdiagnosis as in 76% of the patients with spinal MRI, an unnecessary or insufficient therapy was detected. Two patients that underwent spinal surgery had abnormalities that were believed to be the cause of the problems complained of or had problems that were also explained by spinal pathology.

Another reason for mistreatment is the result of a priorly wrong diagnosis. We found that 26.3% of the patients underwent surgery under an incorrect diagnosis. This includes differential diagnoses such as lipoma, malignant lymph node, or a soft tissue tumor.

The misinterpretation of a symptom and/or the radiological imaging may lead to unnecessary or inappropriate surgical treatment unrelated to the actual disease or to a more radical surgical treatment [25].

This resulted in severe neurological deficits that had to be treated with nerve interposition devices during revision surgery. In the remaining patients, revision surgery was also performed to remove remaining tumor tissue or to perform neurolysis.

Furthermore, a CT-guided biopsy bears the risk of nerve-damaging and is a permanent cause for discussion between the different disciplines. Nevertheless, biopsy or macroscopic removal is performed for diagnostic purposes according to the guidelines of the different oncological, gynecological, or visceral surgical societies [26–28].

Complete resection of schwannomas is standard of care and possible without loss of function [29–32]. Preservation of function must be the primary goal of surgery, especially if these benign tumors have not caused any neurological deficits before surgery [4, 31, 33]. After complete, subcapsular removal of the schwannoma, recurrence is very rare [9, 31].

Removal of a neurofibroma can be more challenging for the surgeon as these often do not have a clear capsule, and injury of the fascicles is more likely [4]. This makes it even more relevant to use a consistent microsurgical technique in these procedures, supported by a surgical microscope, stimulating forceps and, the appropriate microsurgical instrumentation to avoid unnecessary postoperative neurological damage [34–36].

The treatment of perineuriomas has changed over time. A biopsy of a thickened and non-functional fascicle including an epineurotomy is more and more common, due to the slowly progressive course and the lack of delimitation. Complete removal is not recommended, as this would ultimately necessitate nerve replacement with poor neurological outcomes [10, 11, 37].

However, the correct diagnosis did not lead to sufficient therapy in several cases. In our analysis, 26.3% of the patients were treated insufficiently, or surgical treatment was not performed in the recommended manner. Treatment by an experienced nerve surgeon showed significantly lower rates of mistreatment compared with treatment by other specialists. Performing a closed biopsy to confirm the diagnosis of a benign nerve tumor should be the exception and not the norm to avoid unnecessary nerve injury [38].

### Approach for optimization in clinical management

**Fig. 3:**
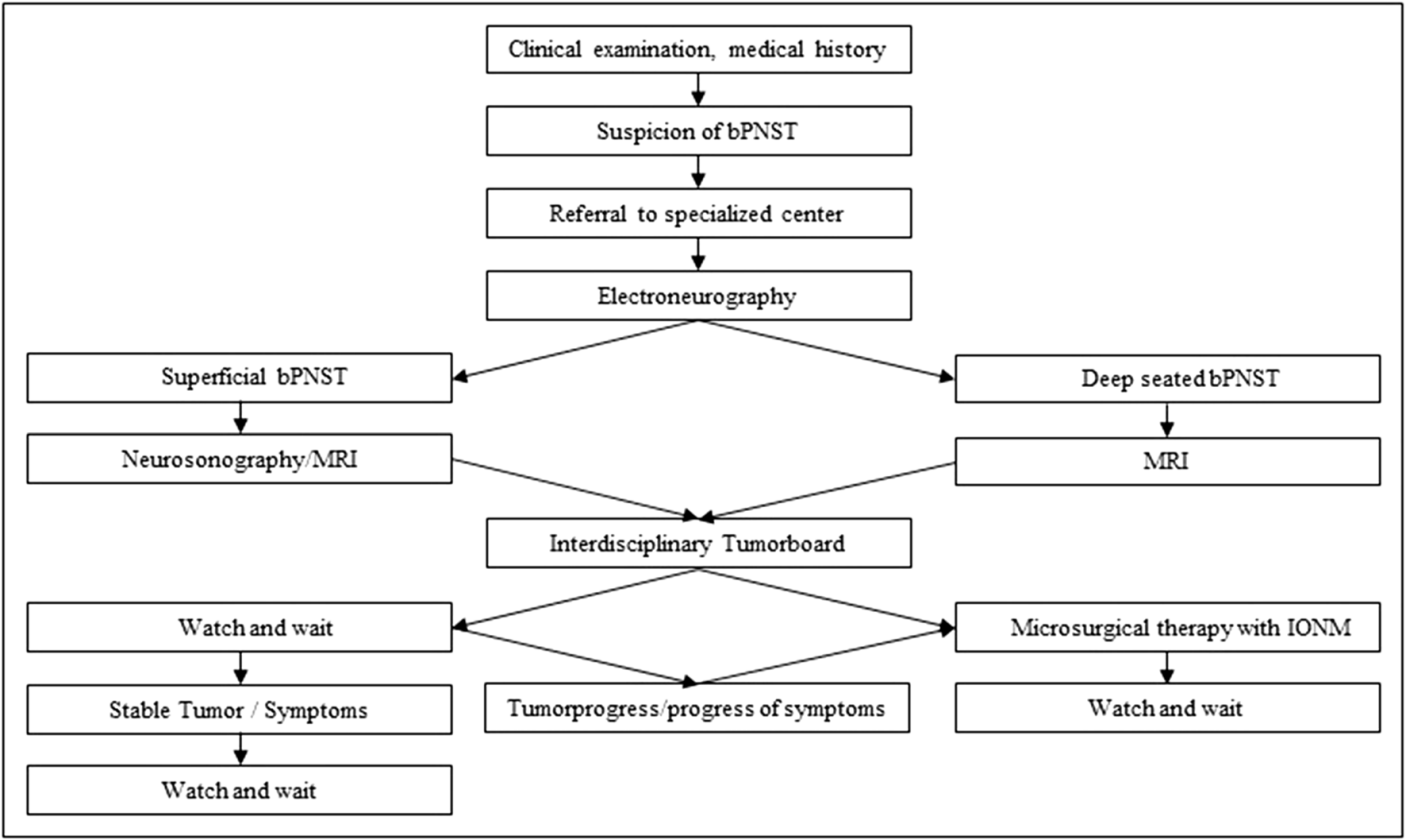
Flow-chart: diagnostical and therapeutical approach of treatment of bPNST. bPNST, benign peripheral nerve sheath tumor; IONM, intraoperative neuromonitoring

Our approach for optimization is based on our experience (Fig. 3). Neurological examination and its interpretation have to be performed with care. It is an important part of diagnosis. Our data supports the relevance of the clinical examination and the Tinel’s sign as an excellent diagnostic tool for the diagnosis of bPNST [39].

Furthermore, the range of diagnostic tools should be widened in daily practice. High-resolution nerve sonography is a promising and straightforward tool to diagnose superficial bPNST, while MRI should be performed in cases of deep-seated bPNST [40–43].

Practitioners should be aware of bPNST as differential diagnosis in patients presenting with a mass located nearby a peripheral nerve, or in patients presenting with neuropathic pain or sensory deficit in a peripheral nerve supply area, and/or motor deficits [44].

Thereupon, interdisciplinary discussion and management in a specialized center may reduce the rate of misdiagnosis and inappropriate treatment. Additionally, rising number of interdisciplinary guidelines may help to keep this rare entity in mind and, therefore, to reduce the number of misdiagnosis and mistreatment in the future.

### Limitations

The present study has several limitations that must be acknowledged. The data were retrospectively analyzed from our prospective dataset “peripheral nerve lesion.” Furthermore, the number of patients included at a single center was limited due to the rarity of bPNST. Therefore, statistics might be influenced by the small sample size and collection bias of a specialized center of peripheral nerve surgery. The German medical system is different from other countries, and the results may not be representative for other regions.

## Conclusions

Tumors of peripheral nerves are commonly benign and can be removed without functional deficits in most cases. However, there is a high risk of initial misdiagnosis due to their rarity leading to a consecutive mistreatment with potential severe consequences. Therefore, treating disciplines have to keep these rare differential diagnoses in mind. Treatment should be performed only at a specialized center.

## Data Availability

Data are available upon request.

## Abbreviations

bPNST: benign peripheral nerve sheath tumor
CT: computed tomography
HNST: hybrid nerve sheet tumor
IONM: intraoperative neuromonitoring
MRI: magnetic resonance imaging
